# A Biophysical Model for Identifying Splicing Regulatory Elements and Their Interactions

**DOI:** 10.1371/journal.pone.0054885

**Published:** 2013-01-30

**Authors:** Ji Wen, Zhibin Chen, Xiaodong Cai

**Affiliations:** 1 Department of Electrical and Computer Engineering, University of Miami, Coral Gables, Florida, United States of America; 2 Department of Microbiology and Immunology, University of Miami, Miami, Florida, United States of America; University of South Florida, United States of America

## Abstract

Alternative splicing (AS) of precursor mRNA (pre-mRNA) is a crucial step in the expression of most eukaryotic genes. Splicing factors (SFs) play an important role in AS regulation by binding to the *cis*-regulatory elements on the pre-mRNA. Although many splicing factors (SFs) and their binding sites have been identified, their combinatorial regulatory effects remain to be elucidated. In this paper, we derive a biophysical model for AS regulation that integrates combinatorial signals of cis-acting splicing regulatory elements (SREs) and their interactions. We also develop a systematic framework for model inference. Applying the biophysical model to a human RNA-Seq data set, we demonstrate that our model can explain 49.1%–66.5% variance of the data, which is comparable to the best result achieved by biophysical models for transcription. In total, we identified 119 SRE pairs between different regions of cassette exons that may regulate exon or intron definition in splicing, and 77 SRE pairs from the same region that may arise from a long motif or two different SREs bound by different SFs. Particularly, putative binding sites of polypyrimidine tract-binding protein (PTB), heterogeneous nuclear ribonucleoprotein (hnRNP) F/H and E/K are identified as interacting SRE pairs, and have been shown to be consistent with the interaction models proposed in previous experimental results. These results show that our biophysical model and inference method provide a means of quantitative modeling of splicing regulation and is a useful tool for identifying SREs and their interactions. The software package for model inference is available under an open source license.

## Introduction

A key step in eukaryotic gene expression is to remove introns from precursor messenger RNA (pre-mRNA) so that exons can be joined together to form the mature mRNA. By including different exons in the mRNA, alternative splicing (AS) can generate different isoforms from a single gene to increase proteomic diversity. Recent studies have found that ∼95% of human genes undergo AS [1], [2]. The importance of AS is highlighted recently by the findings that AS related mutations can cause many human diseases including cancer [3], [4].

AS can be regulated via several mechanisms, often in a tissue-specific manner [5], [6]. One mechanism is that recognition of splice sites by the spliceosome is influenced by a class of RNA binding proteins named splicing factors (SFs) that can bind to the *cis*-acting splicing regulatory elements (SREs) on the pre-mRNA. These SREs are categorized as exonic splicing enhancers (ESEs) and silencers (ESSs), and intronic splicing enhancers (ISEs) and silencers (ISSs) based on their locations and effects on splicing [7]. Recently, several experimental [8]–[10].and computational methods have been employed to identify SREs. The computational methods can be largely categorized into three approaches. The first enrichment-based approach is to identify SREs as short nucleotide sequences (typically hexamers or octamers) that are statistically enriched in a carefully selected set of introns and exons against a background or negative dataset [11]–[13]. The second conservation-based approach utilizes comparative genomic methods to identify evolutionarily conserved motifs in introns and exons, which can also be combined with the enrichment-based approach to identify SREs [14]–[17]. The third regression-based approach exploits both sequence information and expression levels of different isoforms in a unified framework [18], [19]. Comparing with the other two approaches, the regression-based approach offers flexibility of identifying combinatorial regulatory effects of multiple SREs. However, the current regression methods for AS were not developed systematically from a theoretical base, which may limit their performance.

Multiple SREs could act cooperatively to promote or repress splicing by regulating exon or intron definition [20], [21]. However, given the huge number of possible pairs of SREs in different regions, experimental approaches for identifying SRE pairs is expensive and time-consuming if feasible. For this reason, computational methods become an important means of selecting candidate SRE pairs in a systematic and high-throughput manner. Several recent computational works have studied cooperative SRE pairs in AS regulation. Ke and Chasin [22].searched for frequently co-occurred SRE pairs from two ends of exons that mediate exon definition. Friedman *et al.* [23] identified cooperative SRE pairs from two ends of human and mouse introns possibly mediating intron definition. Suyama *et al.* [24] analyzed conserved pentamers that often co-occur in the same region of upstream or downstream introns, which may arise from cooperative binding of different SFs or actually from a single long motif. These different types of SRE pairs from different regions reveal that cooperative interaction between SREs may be a common mechanism in AS regulation. However, these SRE pair detection methods did not incorporate expression data into the analysis, and like the enrichment-based approach to the detection of single SRE, they could not exploit sequence and expression data in a systematic way, which may limit their detection power.

In this paper, we first derive a novel biophysical regression model for the regulation of AS, which can capture both main effects of individual SREs and combinatorial effects of multiple SREs. We then develop a systematic framework to infer the regression model, which in turn identifies both single SREs and different types of cooperative SRE pairs. The key feature of our model inference framework is that we employ the shrinkage technique [25] to identify a small number of SREs and SRE pairs from a huge number of all possible SREs and their pairs. Our numerical results show that our regression model can explain a significant portion of the variance in the data comparable to the best result for transcription achieved by a nonlinear biophysical model [26]. Using an RNA-Seq data set [1], we identify 619 SREs and 196 SRE pairs, some of which are verified with previous experimental results. For example, binding sites of PTB in upstream and downstream of introns of alternatively spliced exons (ASEs), binding sites of hnRNP F/H in two ends of downstream intron, and binding sites of hnRNP E/K in the same region are identified as interacting SRE pairs, which are consistent with existing experimental evidences.

## Results

### Biophysical Model for AS Regulation

The basal machinery of splicing is known as the spliceosome, a large multicomponent ribonucleoprotein complex having U1, U2, U4, U5 and U6 snRNPs as its main building blocks [27]. Splicing begins with a multi-step process of spliceosome assembly around the splice sites and the branch point. SFs bound to nearby SREs can influence spliceosome assembly by facilitating or inhibiting the subunits of spliceosome to recognize the splice sites [7], [28], [29]. They can also regulate splicing through other mechanisms such as regulation of the transition from exon definition to intron definition [5], [30]. Moreover, multiple SFs and the spliceosome can interact cooperatively or antagonistically to affect the splicing process.

**Figure 1:**
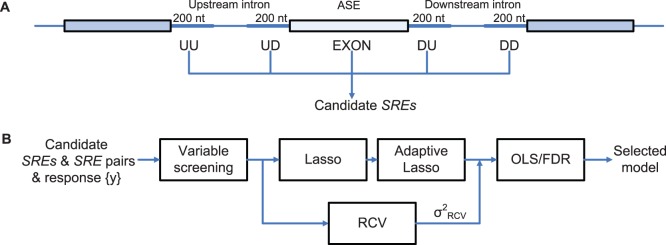
Five regions around ASEs used to extract SREs and the model inference framework. (**A**) All hexamers in the five regions around ASEs are considered as candidate SREs. UU (upstream/upstream) stands for the 5′ end of the upstream intron. UD (upstream/downstream) denotes the 3′ end of the upstream intron. DU and DD are defined in a similar way. EXON stands for the ASE region. (**B**) The inference framework for detecting active SREs and SRE pairs. RCV: refitted cross-validation; OLS: ordinary least squares regression.

We model the spliceosome assembling process with a chemical reaction:
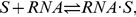
(1)where S denotes the spliceosome. This simplification is similar to the one used in the derivation of a biophysical model [26], [31], [32] for transcription where assembly of the RNA polymerase (RNAP) complex is simplified to one reaction. If an SF binding to an SRE interacts with the spliceosome, it increases or decreases the equilibrium constant of the above reaction, which in turn enhances or inhibits splicing. Let us consider a gene with one ASE and let 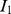 (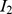) be the isoform that includes (excludes) the ASE. Let 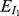 and 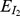 be the expression levels of 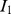 and 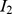, respectively. We assume that the pre-mRNA of the gene with assembled spliceosome produces 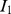, whereas the pre-mRNA without assembled spliceosome produces 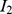. Therefore, the probability of producing 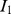 is equal to the probability that the pre-mRNA is bound by the spliceosome. The first probability can also be expressed as 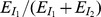, while the second probability can be derived from the biophysical chemical reactions modeling spliceosome assembly and binding of SREs to the pre-mRNA as detailed in Materials and Methods. Based on this observation, we derive the following regression model in Materials and Methods to capture the regulatory effects of SREs on the splicing of an ASE:
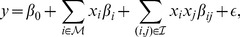
(2)where 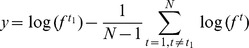, with 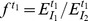 for tissue 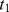 and 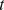 being the index of 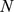 tissues; 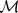 and 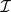 are the set of potential SREs and the set of potential SRE pairs, respectively; 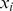 is a binary variable to indicate presence (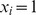) or absence (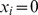) of the 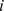
th SRE; 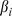 reflects the contribution of the 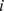
th SRE to the splicing of the ASE, and 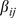 indicates the cooperative contribution of the 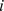
th SRE and the 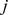
th SRE; 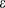 is the measurement error, which is modeled as a Gaussian random variable with zero mean. Note that the 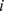
th and 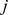
th SREs in the third term at right hand side of (2) can be from the same region or two different regions as described in Figure 1A. The first term 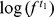 in 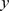 is the logarithm of the ratio between the expression levels of two isoforms including or excluding the ASEs; it is determined by the basal splicing level and the regulatory effects of SFs binding to the SREs. The second term in 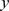 is added to remove the basal splicing level determined by the spliceosome alone as described in Materials and Methods. Thus, given the splicing profile 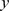 of a set of ASEs and a set of candidate SREs, we can identify SREs or SRE pairs that have regulatory effect by finding 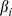 or 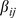 that are not equal to zero with certain statistical significance. The method for model inference to determine 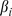 or 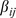 is presented in next section. The condition to determine if an SRE is a enhancer or silencer based on the sign of 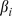 and the expression level of the SF binding to the SRE is given in Proposition 1 in Materials and Methods. Whether an interacting pair of two SREs is an enhancer or silencer, however, is difficult to be inferred from the sign of 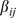.

Note that model (2) is linear with respect to unknown parameters 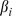 and 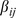. The linearity facilitates model inference even when the number of parameters is very large. As shown in Materials and Methods, linear model (2) is directly derived from biophysical principles. In contrast, the biophysical model for gene transcription [26], [31]–[33] is a nonlinear model with respect to unknown parameters to be inferred. While it is possible to infer parameters of the nonlinear model when the number of parameters is small [26], model inference becomes difficult when the number of parameters is relatively large. The linear regression models for transcription [34]–[37] or for splicing [18], [19] are an approximation of the nonlinear biophysical models, as shown in [36]. Although these linear models enable efficient model inference, their performance is limited by the approximation inherent to the models. A nonlinear model based on regression spline [38] was also developed to approximate the nonlinear biophysical model. Comparing with linear regression, regression spline reduces approximation error, but increases the complexity of model inference.

### Framework for Model Inference

We applied the biophysical model to identify SREs and SRE pairs involved in alternative splicing. As described in Materials and Methods, We first determined a set of ASEs from the UCSC KnownGene table [39], then extracted all the hexamers in five regions around ASEs as candidate SREs (Figure 1A), and obtained 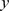 for this set of ASEs from the RNA-Seq data. With this data set, model inference was carried out using four components described in Figure 1B.

Since we considered all 6-mers and their interactions, the regression model (2) contained 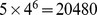 variables for main effects of possible SREs and 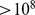 (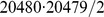) variables for interactions of possible SRE pairs. The huge number of variables not only requires huge computation for model inference, but also may yield a large number of false SREs. To overcome these problems, we developed the four-component framework in Figure 1B to select reliable SREs without overfitting the model. In the first variable screening component, we used the sure independence screening method [40] described in Materials and Methods to remove 6-mers that have very small correlation with the response variable 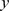.

In the second component, we adopted the Lasso [41] and the adaptive Lasso [42] to perform penalized multiple regression to select variables. Descriptions of the Lasso, the adaptive Lasso and the cross-validation procedure for determining the parameter 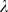 of the Lasso and the adaptive Lasso are given in Materials and Methods. The Lasso is known to shrink many variables, with no or small correlation with the response variable, to zero [41], and thus yields a sparse model that only contains a small number of variables. Using both the Lasso and the adaptive Lasso was to ensure more reliable variable selection as suggested in [43]. This component produced a sequence of models for different values of 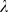. The minimum variance of the residual error at 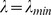 determined by cross-validation was denoted as 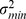.

Although the Lasso and the adaptive Lasso only retained a small number of variables in the model, we wanted to ensure that the overfitting problem did not occur. To this end, we added the third component named refitted cross-validation (RCV) [44] to the inference procedure. RCV reliably estimates the variance 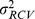 of the residual error in a linear model of ultrahigh dimension. We then compared 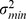, with 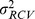. If 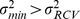, we selected variables that gave 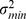; otherwise, we identified the value of 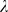 that yielded a residual variance equal to 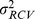 and selected variables with this 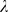.

The adaptive Lasso together with RCV selected a set of SREs and SRE pairs, but it did not give p-value for each variable in the model. In the last component, we used ordinary least squares (OLS) method to refit the model with the variables selected by the adaptive Lasso. We then used the p-values provided by OLS to select variables at a false discovery rate (FDR) 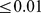 [45]. This final set of variables were identified as SREs and SRE pairs. A software package implementing the inference framework is freely available under an open source license.

### Performance of the Model and the Regression Framework

As described in Materials and Methods, we selected a set of ASEs for each tissue from the KnownGene table and calculated the inclusion ratio 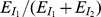 from the RNA-Seq data [1] for each ASE, which was then used to calculate the response 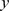 in model (2). We applied our biophysical model and inference framework to this data set to identify SREs and SRE pairs. The number of ASEs used in model inference, the number of SREs and SRE pairs in the final model and the percentage variance explained by the final model are given in Table 1. In each tissue, our biophysical model explained 49.1%–66.5% of the variance in the data (see 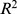 in Table 1), which was comparable to that achieved by the best model for transcription reported in [26]. More specifically, the linear model for gene transcription in [34] explained 9.6% of the variance on average, while the regression spline model for gene expression that incorporated interaction terms explained 13.9% to 32.9% of the variance [38]. Most recent work by Gertz *et al.* [26] fitted a nonlinear biophysical model to the expression data of synthetic genes. Their models explained 44–59% of the variance in gene expression. Thus, considering the non-synthetic genes we used, our model has captured a large fraction of the variance in the splicing response.

**Table 1:** Summary of the final selected models in each tissue.

Tissue	No. of ASEs	No. of SREs	No. of SRE pairs	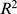 (%)
Adipose	1345	60	30	52.9
Brain	1411	65	19	49.1
Breast	1399	82	24	58.3
Colon	1236	64	13	49.4
Heart	1302	84	21	60.5
Liver	995	76	19	66.5
Lymph node	1405	77	24	55.7
Skeletal muscle	1174	85	18	63.2
Testes	1601	79	28	51.0

The second column is the number of ASEs used in model inference. The third and fourth columns list the number of SREs and SRE pairs in the final model. The last column gives the percentage of the variance explained (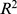) by the final model.

Overall, 619 different SREs and 196 SRE pairs were detected from different tissues. Specifically, Table S1 contains all the SREs and SRE pairs identified from different tissues, regression coefficient and p-value for each SRE or SRE pair. It also includes some SFs that have experimental evidence (SELEX [46]–[65], RNAcompete [66] and other experiments [67]–[75]) to bind to the identified SREs. These SREs and SRE pairs consist of 854 different hexamers. Many SREs are very similar to each other, which may arise from an SRE longer than 6 nucleotides (nts) or from a degenerate motif. Note that although they were detected in different tissues, the SRE and SRE pairs *per se* do not give rise to tissue-specific isoforms, because they are always there in pre-mRNA sequences in different tissues. It is the tissue-specific expression of SFs that regulates tissue-specific splicing through SREs.

We compared our 854 SREs with the results of Castle *et al.* [12], who performed a systematic screening of all 4 to 7-mers for cis-regulatory motifs enriched near ASEs using microarray. We only kept 5 to 7-mers with a p-value smaller than 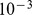 (Bonferroni-corrected p-values as described in [12]) for the comparison. In total, 137 non-redundant 5 to 7-mers were left (91 5-mers, 28 6-mers and 18 7-mers). We considered it as a match if a 7-mer contains one of our SREs, a 5-mer is part of our SREs, or a 6-mer exactly matches one of our SREs. This yielded 103 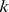-mers, 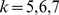, that could find at least one match in our SREs. In order to evaluate the significance of the overlap, we generated a list of 6-mers from the 137 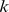-mers of Castle *et al.* with the following procedure. For each 5-mer, 8 different 6-mers containing the 5-mer were obtained by padding a nucleotide to the beginning or the end of the 5-mer. For each 7-mer, 2 different 6-mers were obtained by extracting the first or the last 6 nts. In total, 639 different 6-mers were extracted from Castle’s 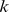-mers, 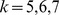. A significant number of 6-mer (180) were found in both our 854 SREs and the 639 6-mers obtained from 137 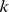-mers of Castle *et al.* (p-value = 9.37e−7 from Fisher’s exact test).

We also compared our 854 SREs with the binding sites of 25 SFs experimentally identified with SELEX [46]–[65] or RNAcompete [66]. Each of [46]–[65] attempted to determine the binding sites of 1 to 3 SFs using SELEX, and [46]–[65] reported SELEX results for a total of 25 SFs. For each of 25 SFs, we obtained a set of RNA sequences selected with SELEX from one of [46]–[65]. If there are more than one SELEX results for an SF, we used the most recent SELEX result. We then extracted the consensus sequences embedded in this set of RNA sequences as the binding sites of the SF. If a consensus binding site is 4 or 5 nt long, one more nucleotide was also extracted from each side of the original selected sequence to obtain hexamers. If a consensus binding site is longer than 6 nts, all the hexamers included in the consensus were extracted. For RNAcompete, the 7-mers listed in Figure 2 of [66] were used as consensus binding sites, and two hexamers were taken from each 7-mer. In total, 709 different hexamers were obtained from the consensus binding sites. A significant number (175) of hexamers were found in both our 854 SREs and the 709 hexamers obtained from SELEX or RNAcompete (p-value = 0.004 from Fisher’s exact test). In Table S1, we gave the SF that was identified in [46]–[66] to bind to one of the 175 hexamers. Although the overlap between our predicted SREs and the binding sites of SFs determined with SELEX and RNAcompete is statistically significant, a relative large number (679) of our predicted SREs are not included in these experimental results, which implies that our result contains some novel SREs.

**Figure 2:**
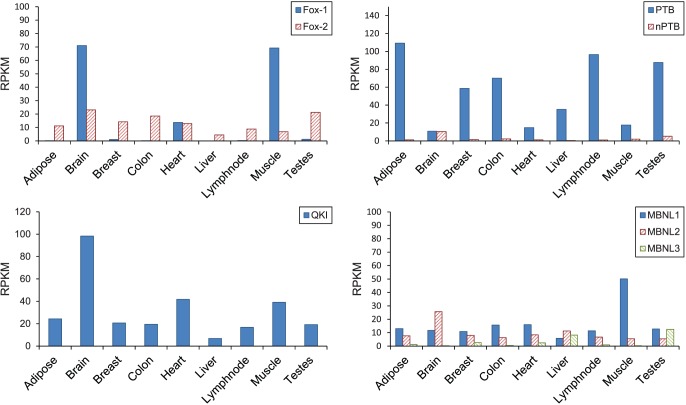
Expression level of several splicing factors in 9 tissues. The expression levels are calculated from the RNA-Seq data [1] as reads per kilobases per million mapped reads (RPKM).

### Experimental Evidence of SREs

Several well-defined SREs involved in tissue-specific AS have been detected in our work. We will take SREs bound by Fox-1 protein, polypyrimidine tract binding protein (PTB), quaking protein (QKI), muscleblind-like protein (MBNL) as examples (listed in Table 2) to illustrate how to understand the results and compare them with the available experimental evidences.

**Table 2:** Selected examples of the detected SREs.

Putative SF	SRE(region)	P-value	# Occ.	Tissue	Effect
Fox-1	UGCAUG(DU)	7.02E−13	98	muscle	enhancer
Fox-1	UGCAUG(UU)/GCAUGU(UU)	1.34E−3	28	heart	unknown
PTB	CUCUCU(UD)	9.76E−6	140	lymph node	silencer
PTB	UUCUCU(UD)	9.33E−4	201	adipose	silencer
QKI	ACUAAC(UD)	3.36E−8	39	muscle	silencer
MBNL	UGCUGC(UU)	1.22E−6	110	lymph node	unknown
MBNL	UGCUGC(EXON)	2.61E−3	65	muscle	unknown

# Occ. is abbreviation for number of occurrences.

Fox protein recognizes [U]GCAUG as its SRE and it has been shown to be one of the most conserved regulators of tissue-specific AS in metazoans. Fox-1 is exclusively expressed in brain, heart, and skeletal muscle as reported in [67], which is consistent with the RNA-Seq data we used as shown in Figure 2. Its paralog Fox-2 has relatively low expression level in all tissues. In our results, we detected two SREs containing UGCAUG as summarized in Table 2. These 2 SREs were detected in heart or muscle, which is consistent with the tissues where Fox-1 is expressed. Note that the second SRE UGCAUG(UU)-GCAUGU(UU) detected in upstream region in heart is an interaction term in the regression model. Since our model includes interaction between SREs in the same region or from different regions, it is possible that an SRE longer than 6 nts is detected as an interaction term. This interaction term actually arises from a 7-mer SRE UGCAUGU(UU) [68] (Among the 28 UGCAUG(UU)-GCAUGU(UU) pairs used for inference in heart, 27 are derived from UGCAUGU(UU)). Using the inference method for regulatory effects described in Materials and Methods, we found that the SRE that we identified from muscle are enhancers in the downstream region (DU) (Table 2), which is consistent with the computational analysis [1] and the experimental evidence [67], [76].

Another well-defined SF is PTB which binds to UC-rich SREs and has high binding affinity to UCUU and/or UCUCU [69], [77]. Two SREs we identified are possible binding sites of PTB (Table 2). They are found in the upstream region in adipose and lymph node. Both SREs are detected from the tissues where PTB are over-expressed as shown in Figure 2. Using Proposition 1 in Materials and Methods, These two SREs were determined to be a silencer in the upstream of ASEs. This result coincides with position-dependent alternative splicing activity of the PTB as identified using microarrays [78].

One of QKI’s binding site ACUAAY [59], [70] was detected in the upstream intron of the ASE in muscle (Table 2). The SRE ACUAAC was previously reported as an over-represented motif in the downstream region of ASEs in muscle [18] and was also predicted as an upstream intronic SRE specific to the central nervous system [79]. Our result indicates that this upstream SRE detected in muscle is a silencer, which is consistent with the experimental result [80].

The MBNL family protein can bind to the motif YGCU(U/G)Y [71]. Two sequences of this motif can be found in our SREs (Table 2). However, the function of this motif is complicated. First, both MBNL and CELF family proteins can bind to similar motifs [81], [82], although different protein isoforms may have different binding specificities [83]. Second, MBNL can either promote or repress splicing of specific ASEs on different pre-mRNAs by antagonizing the activity of CELF proteins [71]. Thus, although our result indicates that this SRE is related to AS regulation in muscle and lymph node, it is difficult to interpret its regulatory effect based on current result.

### Experimental Evidence of SRE Pairs

**Figure 3:**
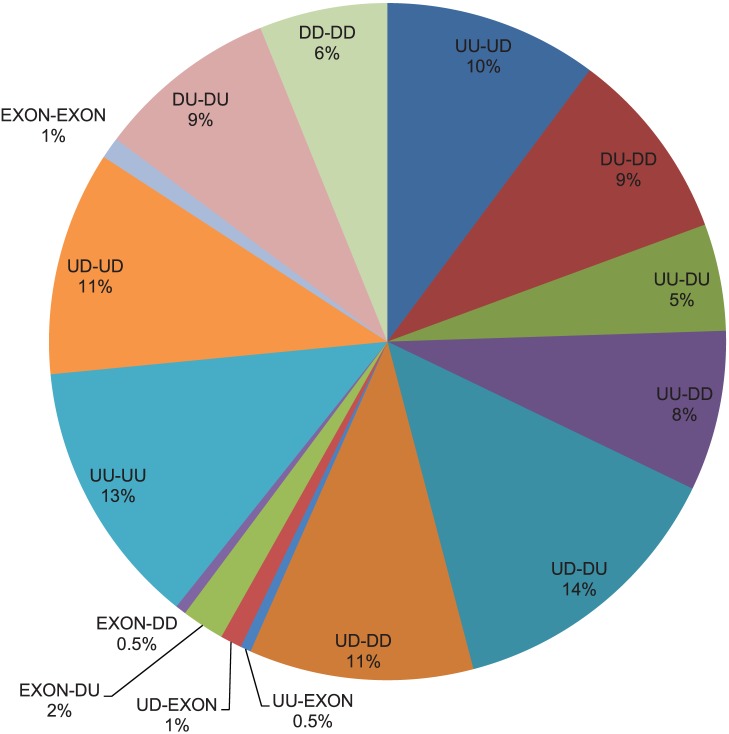
Percentage of different types of SRE pairs. Two hundred and forty-one SRE pairs are detected in different or same regions. This figure shows breakdown of the SRE pairs in different regions.

We also identified 196 different cooperative SRE pairs (Table S1). Thirty-nine percent (77 SRE pairs) of them are interactions of SREs in the same region. As discussed in the previous section, a part of this kind of interaction may come from a single SRE longer than 6 nts. These SRE pairs are actually not related to interactions, although they also have biological meanings, since a longer conserved SRE may have stronger regulatory effect than a shorter SRE. We have marked all 23 such SRE pairs in Table S1. Among the remaining interactions between different regions (119 SRE pairs), the most frequent interactions are SREs in region pairs UD-DU, UU-UD and UD-DD. Region pair UD-DU may reflect the effect in the exon definition stage of spliceosome assembly, and region pair UU-UD may reflect the effect in the intron definition stage. A summary of interaction between SRE pairs in different regions is given in Figure 3. Several detected SRE pairs are listed in Table 3.

**Table 3:** Selected examples of the detected cooperative SRE pairs.

Putative*SF*_1_	*SRE*_1_(region)	Putative*SF*_2_	*SRE*_2_(region)	P-value	Tissue
PTB	UUCUCU(UD)	PTB	UCCUCU(UD)	2.02E−4	brain
PTB	UCUUCC(UD)	PTB	CCUUCU(DD)	1.02E−6	brain
hnRNP F/H	GGGGCA(DU)	hnRNP F/H	UGGGGA(DD)	1.31E−3	liver
hnRNP E/K	CCCCAG(UU)	hnRNP E/K	CCGCCC(UU)	8.02E−8	lymph node
hnRNP E/K	ACCCCU(DU)	hnRNP E/K	CCCCUC(DU)	8.70E−4	adipose
T/H/S	AUUUAU(UU)	T/H/S	UAAAUG(UD)	2.33E−10	brain
T/H/S	AUUUAC(DU)	T/H/S	AAUAAA(DD)	3.04E−9	lymph node
T/H/S	AAAUUU(UD)	T/H/S	UUUUUU(DU)	3.71E−7	lymph node
T/H/S	AAUAUG(UU)	T/H/S	AAAAAU(UD)	3.75E−5	heart
T/H/S	AUUUAG(UD)	T/H/S	UUAUAU(DU)	1.02E−4	testes
T/H/S	UUUAAU(UD)	T/H/S	UUUUAU(DD)	1.45E−4	adipose
T/H/S	AAAUUC(UU)	T/H/S	AAAUUU(DU)	6.08E−4	lymph node
T/H/S	UUUUAA(UD)	T/H/S	CAUUAU(DU)	8.93E−4	adipose
T/H/S	UUUAAU(UU)	T/H/S	AAAAUA(UD)	1.09E−3	heart
T/H/S	GUUUUA(UD)	T/H/S	UUUUAU(DD)	1.90E−3	adipose
T/H/S	AAUAUU(UD)	T/H/S	AUUUUG(DD)	3.06E−3	breast
T/H/S	UAUUUA(UD)	QKI	AACUAA(DU)	2.47E−6	liver
T/H/S	AUUUAA(UU)	PTB	CUUUUC(UD)	6.12E−4	heart

T/H/S represent TIA-1/TIAR, Hu family or Sam68 proteins.

Several previously identified SREs were detected in our interaction results. They either cooperate with their own or other different SREs in a different region or in the same region. Two SRE pairs bound by PTB [69], [77] were identified in our result. All the four SREs located at the upstream region of the 3′ splice site which can also be a part of an extended polypyrimidine tract, although the 3′ splice site consensus of 15 nts containing the classical polypyrimidine tract [7], [11] has been removed in our analysis. One SRE pair UUCUCU-UCCUCU was identified in the upstream intron in brain. The other interaction detected involves PTB’s binding site UCUUCC in the UD region and CCUUCU in the DD region (Table 3). The downstream CCUUCU resembles the PTB binding sites and might also be bound by PTB. In a cooperative model for the regulatory mechanism of PTB, it was proposed that a single PTB or a PTB dimer could loop out the branch point upstream of 3′ splice site or the ASE by binding to several pyrimidine tracts upstream and downstream to repress splicing [84]. A single PTB-binding element had weak silencing activity, while multiple PTB-binding sites at both upstream and downstream of the ASE or of a branch point could have strong inhibitory effect [85]. This model and the experimental results in [84], [85] are consistent with the interaction pairs we identified.

hnRNP F/H can bind to GGGA and G-rich SREs [72], [73]. The proposed mechanism underlying the splicing regulation of hnRNP F/H involves an interaction between proteins bound at both ends of an intron that loops out the intron and brings distantly separated exons into closer proximity [86]. Consistent with this model, one pair of SREs GGGGCA(DU)/UGGGGA(DD) (Table 3) putatively bound by the hnRNP F/H was detected in two ends of the downstream intron.

Both hnRNP E and hnRNP K proteins contain three copies of the KH domain arranged in a very similar manner, and they are the major poly-C binding proteins in mammalian cells [55], [74]. We extracted all the SRE pairs in our result that contain at least 4 Cs. Two such SRE pairs (Table 3) were found to resemble the binding motif of the hnRNP E/K [55]. One SRE pair is due to a longer motif ACCCCUC at downstream intron. The other SRE pair was detected as interaction in the same region. This result may reflect the fact that the three KH domains bind RNA synergistically while a single KH domain appears to have very low level of RNA binding activity [87].

An interesting outcome of this work was the identification of many AU-rich elements in SREs and SRE pairs. The AU-rich elements have been identified previously by comparative analysis as a large class of conserved mammalian ISEs [17]. In plant splicing system, the AU-rich sequences in upstream and downstream introns appear to be involved in early intron recognition and stabilization of spliceosomal complex [88]; and multiple short AU-rich SREs could cooperatively modulate splice site usage [89]. However, the interaction between AU-rich SREs has not been reported in mammals. In our result, The SRE pairs involved in two AU-rich SREs from different regions are listed in Table 3. Among these SRE pairs, 7 pairs were detected in upstream and downstream introns, and 4 were detected at two ends of the same intron. These AU-rich SREs resemble the binding sites of TIA-1/TIAR, Hu protein and Sam68 [49], [90]. Hu proteins can bind to both upstream and downstream SREs. It has been speculated that binding of Hu protein to multiple sites both upstream and downstream of an ASE could loop out the ASE and as a result block exon definition to repress splicing [90]. Moreover, Hu proteins can promote exon inclusion by binding to AU-rich sequences that are conserved at both upstream and downstream of ASE [91]. The intricate experimental results stress the importance of further mutation analysis to study the cooperative interactions of AU-rich binding proteins.

We also identified many interactions between AU-rich elements and binding sites of other known SFs. For example, We found one interactions between upstream AU-rich SRE and a downstream SRE resembling QKI’s binding site. Another example is the interactions between upstream AU-rich SRE and a CU-rich SRE resembling polypyrimidine tract (Table 3). These results indicate that AU-rich SREs and cooperative pairs may play an important regulatory role in mammalian AS and worth further experimental investigations. In summary, various cooperative mechanisms could be detected in our result as an interaction, which implies the important role of interaction in splicing regulation.

## Discussion

Our regression model for splicing regulation is derived from the same biophysical principle regarding protein and nucleic acids interaction as the one used to derive the model for transcription [26], [31]–[33]. However, our model uses the ratio of the expression levels of two isoforms or equivalently the ratio of binding and unbinding probabilities of the spliceosome to the mRNA as the response variable, whereas the model for transcription uses the gene expression level or equivalently the binding probability of the RNAP to the promoter as the response variable. For this reason, our model for splicing is linear with respect to unknown parameters to be inferred, whereas the model for transcription is nonlinear. While the nonlinear model for transcription with relatively small number of unknown parameters can be directly inferred [26], a linear approximation [34]–[37] and an nonlinear approximation using splines [18], [38] have been proposed to facilitate model inference. It was shown that the spline approximation [37], [38] can offer significantly better performance than the linear approximation in terms of the variance explained by the model. The linear regression model [19] and the spline regression model [18] for splicing regulation use the expression level of an isoform as the response variable. Therefore these two models are also approximate models. In contrast, our regression model is directly derived from the biophysical principle and the linearity of our model with respect to unknown parameters enables efficient model inference even when the number of unknown parameters is very large. This explains why the variance explained by our model is comparable to the best result achieved by the nonlinear biophysical model for transcription [26]. Our model may be improved to explain more variance, for example, by including interactions involved more than two SREs, by accounting for the number of occurrences of each SRE, and by including SREs of length not necessarily equal to six nucleotides, although this can increase the complexity of model inference. On the other hand, if we are interested in the identification of SREs interacting with the binding sites of a specific SF, we can design well-controlled experiments which measure the splicing profile of all the genes before and after knockdown of a specific SF, and then apply our model to identify SRE pairs related to the SF.

To the best of our knowledge, this is the first time that the regression-based approach is employed to systematically identify cooperative SRE pairs. Moreover, our regression framework can identify SRE pairs without being affected by GC content. Current methods for identifying cooperative SRE pairs in splicing regulation use hypergeometric test to find the co-occurrence of SRE pairs over-presented in different regions [22]–[24]. Since they only use sequence information, some sequence features that are not related to splicing regulation may increase the FDR. For example, without correction of GC content, the majority of the motif pairs detected in [22], [23] with high p-values share similar GC contents, being either GC-rich or AU-rich. For this reason, they corrected the GC content by grouping the ASEs with similar GC content [22], [23]. However, If AU-rich or GC-rich SRE pairs indeed have regulatory effects, correction for GC content might introduce bias and under- or over-estimate the statistical significance of the SRE pairs. In fact, it has been shown that the AU-rich motif is conserved in mammalian introns [17] and could be bound by TIA-1/TIAR, Hu protein or Sam68 [49], [90]. In our work, since our model uses the isoform expression information in addition to the sequence information, it can automatically handle the GC content problem. If there is no correlation between the splicing response and the GC- or AU-rich SRE pairs, these pairs will not be identified since they are just sequence features irrelevant to splicing regulation. On the other hand, if they do have regulatory effect, our method will identify them as SRE pairs based on their correlation with the response.

Since we want to detect all possible SREs and SRE pairs, our linear regression model contains a very large number of candidate SREs and their pairs as variables. The challenging problem in model inference is to select correct variables without overfitting the data. We employed four techniques to tackle this problem. First, variable screening is used to exclude SREs and SRE pairs that are present in less than 1% of the samples or have no or very small correlation with the response variable. Second, regularized inference methods, the Lasso and the adaptive Lasso, were employed in conjunction with cross-validation to select a small number of SREs and SRE pairs. Third, RCV is used to estimate the residual variance which was further used to prevent the possible overfitting problem. Finally, the FDR was calculated to retain only the most statistically significant SREs and SRE pairs in the final model. Overall, these steps combine the state-of-the-art techniques and form an effective framework to reduce the FDR and prevent model overfitting, without compromising the power of detection.

The SREs and SRE pairs we identified have a significant overlap with a set of SREs identified with experiments. The regulatory effects of several well-defined SREs were correctly inferred from our model. For several different interaction patterns proposed based on the experimental results, our model successfully identified them as SRE pairs in the same regions as the proposed ones and provided more insight into their interactions. Note that we can identify SRE pairs at two ends of intron [23], at two ends of exon [22], and in the same region [24] in one framework, and capture the combinatorial regulatory effects of multiple SREs more faithfully. We also report AU-rich SRE pairs as a putative interaction pattern that is important and prevalent in human splicing regulation. In summary, our biophysical regression model provides a useful platform for discovering splicing regulators and unraveling splicing regulatory mechanisms.

## Materials and Methods

### The Biophysical Model

Suppose a gene contains an ASE, and it can generate an isoform 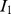 with the ASE or another isoform 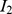 without the ASE. Since splicing is coupled with transcription and the product emerging from this coupled process is either  or 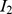, we can consider the splicing of each pre-mRNA independently. We model the multi-step assembly of spliceosome 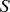 to the splice sites of the ASE on each individual pre-mRNA as a single chemical reaction:
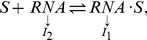
(3)where 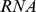 denotes the state in which S is not fully assembled, and 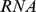 represents the state in which S is fully assembled around the ASE. Then 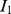 is produced from the 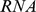 state and 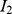 is produced from the 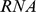 state. The probability of having a spliceosome assembled around the ASE is equal to the probability of the 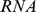 state in reaction (3), which can be expressed as 
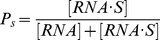
, where [RNA

S], [RNA.and [S.stand for the concentrations of RNA

S, RNA and S, respectively. Since the equilibrium constant of reaction (3) is given by 
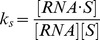
, we can write 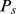 as follows:
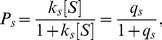
(4)where 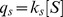. Similar to the gene expression model [36], [38], the dynamic changes of the concentration of 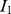 denoted as 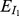 can be written as:
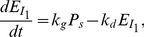
(5)where 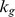 and 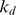 are synthesis and degradation rates, respectively. In the steady state where 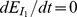, we have 
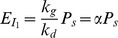
, where 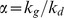. Likewise, concentration of the second isoform 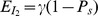, where 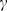 is another constant. Thus, the ratio of 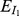 and 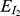 can be written as:
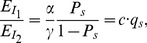
(6)where constant 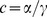. Now we consider an SF that can bind to an SRE around the ASE and influence the assembly of the spliceosome. The pre-mRNA can have four possible states: 1) bound by both S and SF (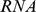), 2) bound by S only (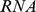), 3) bound by SF only (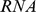), and 4) bound by neither of them (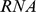). Then the probability 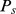 of having a spliceosome assembled around the ASE is equal to the probability of states 1) and 2). Following [26], [31], [32], we can write 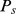 as 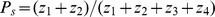, where 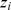 is the Boltzmann weight for state 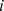. Let 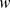 be the cooperative factor reflecting the interaction between SF and S, 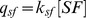 where 
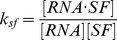
, then it is not difficult to find that 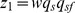, 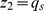, 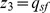 and 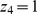, which yields:



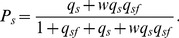
(7)If 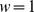, SF and S bind to the transcript independently and 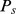 in (7) is simplified to that in (4). If 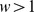, the binding of SF to SRE increases the probability of spliceosome assembly, which implies that the SF is an enhancer. If 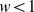, binding of the SF has a negative effect on spliceosome assembly and the SF is a repressor. The ratio of the expression levels of 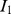 and 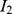 can be written as:
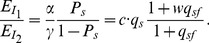
(8)


**Figure 4:**
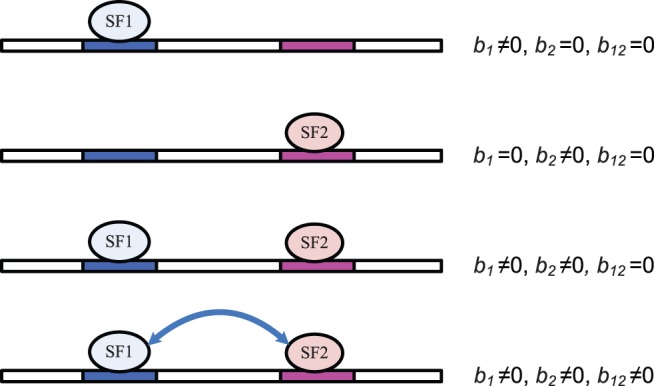
Illustration of equation (12). Two possible SREs are considered in this example, one for SF1, and the other one for SF2. Four different conditions are shown. Binding of either SF can affect the probability of spliceosome assembly. The arrow connecting two SFs indicates the interaction between two SFs. The contribution to spliceosome assembly from SFs is represented by 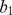, 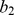 and 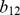.

If two SFs can cooperatively bind to their SREs around the ASE and interact with the spliceosome, it is not difficult to derive the following ratio:
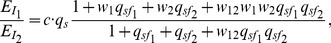
(9)where 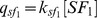 with 
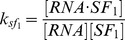
, 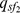 and 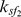 are defined similarly for 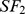, 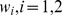, is the cooperativity factor between 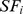 and S, and 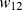 is the cooperativity factor between two SFs. If 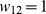, there is no cooperative interaction between two SFs, and they enhance or repress spliceosome assembly independently. In this case, (9) can be simplified as



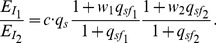
(10)If 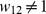, we can express (9) as:
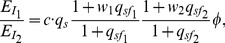
(11)where 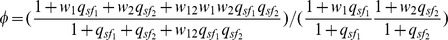. If we define 
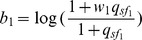
, 
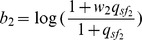
 and 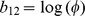, we can write (11) as:



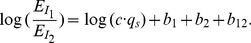
(12)The first term reflects the basal level of splicing determined by the spliceosome alone, the second and third terms are the effects of interactions between the spliceosome and each individual SF, while the last term is the effect of the interaction between two SFs. This can be seen from the fact that 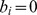 if 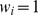 and 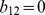 if 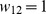. In other words, if the 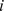
th SRE affects splicing, then 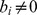; otherwise 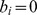; Similarly, if two SREs interact with each other and affect splicing jointly, then 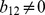; otherwise 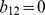. Four different conditions are depicted in Figure 4.

Note that 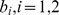, is determined by 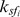, 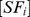 and 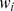. Since an SF can bind to the same set of SREs around different ASEs, we assume that 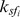 and 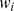 are the same for different ASEs. Therefore, if we consider a set of ASEs in the same tissue or under the same condition, where 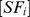 is fixed, 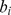 is identical for these ASEs. Similarly, we assume that 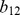 is a constant for different ASEs in the same tissue. On the other hand, since different exons may have strong or weak splice sites and different genes may have different degradation rates, the first term 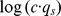 in (12) may be different for different exons even in the same tissue or under the same condition. Since our goal is to infer 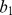, 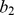 and 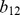 from data of multiple ASEs in the same tissue, we need to remove the exon-specific effects from the model.

If expression levels of isoforms in two tissues 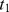 and 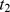 are available, the first term in (12) is identical in these two tissues, and can be removed by forming the following model:
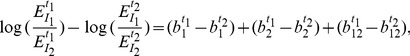
(13)


The data of tissue 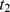 can be regarded as a reference. Subtraction of the reference data from the data of tissue 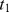 removes the exon-specific effects. When data of multiple tissues are available, we can arbitrarily choose a tissue as the reference. However, since the expression level of each isoform is estimated from the noisy measurements, a better reference can be obtained by averaging the data of multiple tissues, which is similar to the strategy used in [18]. Specifically, suppose we have a set of data 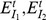 for tissue 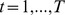, we can remove the first term in (12) by forming the following model:
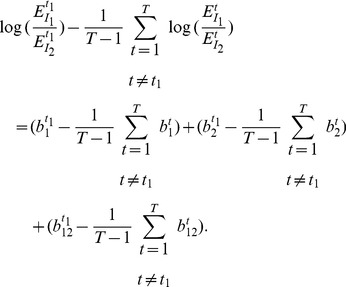
(14)


If we define 
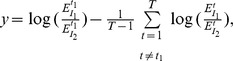
 
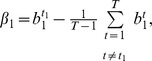
 
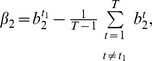
 and 
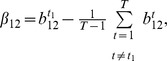
 then (14) can be simplified as:
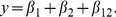
(15)


So far, we have assumed that 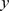 can be measured without any error. If the measurement error is taken into account, equation (15) becomes:
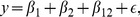
(16)where 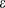 is the measurement error modeled as a Gaussian random variable with zero mean. Model (16) is derived under the assumption that two SREs are present to regulate splicing. Since we do not know which SRE or SRE pair contributes to splicing, we can include all potential SREs and their pairwise interaction in the model by adding a parameter to the model for each potential SRE and SRE pairs. Moreover, when we use this model to identify SREs and their interactions, we need to apply it to a set of ASEs. However, different ASEs may have different SREs. To overcome this problem, we include all possible SREs (typically hexamers) and their pairwise interactions in the model, but multiply 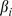 by a binary variable 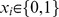 that indicates if the corresponding SRE is present in the 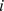
th ASE, and similarly multiply 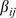 by 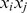. This gives rise to the model in (2). We can also include interactions involving more than two SREs, but this will dramatically increase the number of unknowns that is already very large, which will make model inference extremely difficult if not impossible. For this reason, we only include pairwise interactions in our model.

### Inference of Regulatory Effects

Our model inference framework will infer 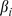 and 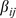 in regression model (2). If 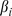 or 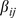 is not equal to zero with certain statistical significance, then we determine that the 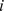
th element or the pair involving the 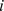
th and 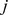
th elements has regulatory effect. However, it is unclear whether it is an enhancer or silencer, since it is the sign of 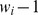 or 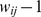, not the sign of 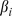 or 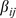 that determines an enhancing or inhibitory effect. We will next show that we can infer the regulatory effect of the 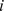
th SRE from 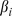 and 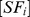 that is the concentration of the SF that can bind to the SRE. The enhancing or inhibitory effect of an SRE pair however is difficult to infer.

Let us first consider the situation where only one reference sample is used as in (13). In this case, 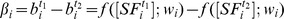, where 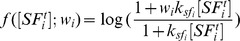. Then it can be easily shown that the sign of 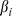 is the same as the sign of 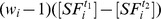. Therefore, if 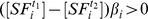, then 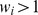 and SRE 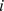 is an enhancer; otherwise, 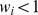 and SRE 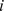 is a silencer.

For model (2) or (15) where multiple tissues are used as the reference, the following proposition can be used to infer the regulatory effect of an SRE:

**Proposition 1** *Given the condition*

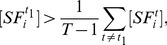
(17)
*we have* 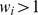 *if* 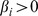, *or* 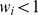 *if* 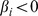. *Given the condition*

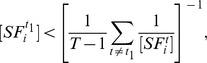
(18)we have 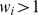 if 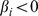, or 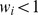 if 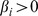.

Before we proceed to prove Proposition 1, we make the following comments. The sign of 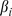 alone can not determine if the SRE is an enhancer (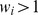) or silencer (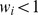). It should be used together with condition (17) and (18) to infer the regulatory effect of the SRE. It is difficult to determine the regulatory effect of an SRE interacting pair bound by two SFs.

**Figure 5:**
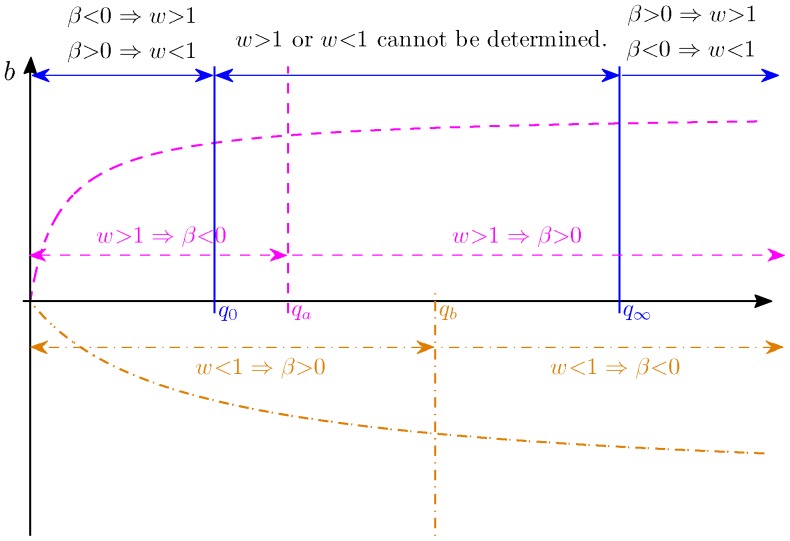
Illustration of Proposition 1. The dashed curve is 
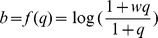
 for 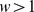 and the dashed lines with arrows define the region where 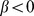 or 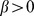. The dash-dot curve is 
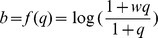
 for 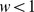 and the dash-dot lines with arrows define the region where 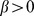 or 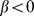. The solid lines define the decision region of 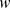 described in Proposition 1.

Proof: For simplicity, we will omit subscript 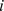 throughout the proof. Define 
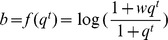
, where 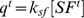 as defined earlier. Then 
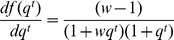
. If 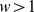, 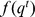 is a monotonically increasing function; otherwise, 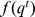 is a monotonically decreasing function (Figure 5).

Define 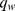 such that 
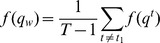
, which gives:
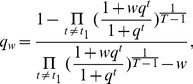
(19)where 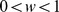 or 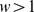. From (19), we obtain the following result:



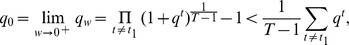


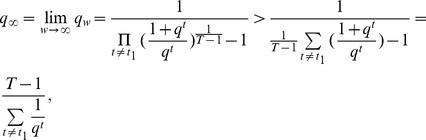



Define 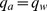 for 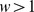 and 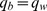 for 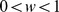. In Lemma 1 given later at the end of this section, we prove that 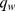 is a monotonically decreasing function of 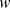. Therefore, we have the following inequalities:
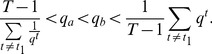
(20)


From (14) and (15), we have 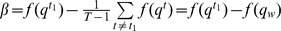. When 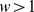, due to the fact that 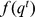 is an increasing function, we have 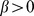 if 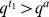 or 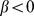 if 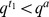. Similarly, when 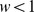, we have 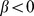 if 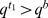 or 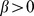 if 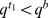. This is illustrated in Figure 5. Now suppose that 
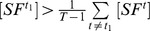
, since 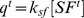, we have 
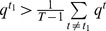
. Using (20), we have 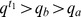. Therefore, we can infer that 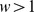 if 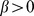, or 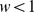 if 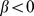 as illustrated in Figure 5. Similarly, if 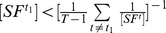, we have 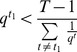, and thus, 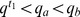. We can infer that 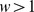 if 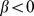, or 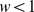 if 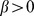, again as illustrated in Figure 5. Note that 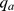 and 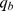 are determined by unknown parameter 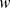 and they can be anywhere in between 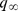 and 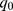. Therefore, if 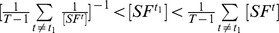, we can not determine whether 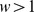 or 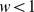.

**Lemma 1**

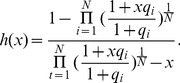
(21)
*is a monotonically decreasing function for* 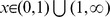, *when* 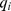, 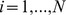 *are positive*.

Proof: Define 
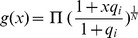
. Then 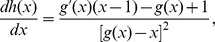 where 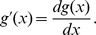 Let us define the numerator of 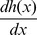 as 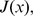 since the denominator is positive, we next prove that 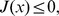 which implies that 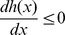 and therefore 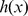 is a decreasing function.
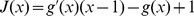


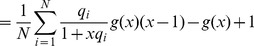


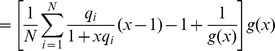


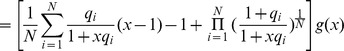


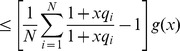


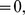
where the inequality is due to the facts that 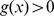 and that 
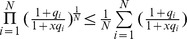
, since 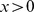 and 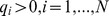. Thus, 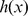 is monotonically decreasing in 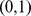 and 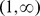. Moreover, since we have:



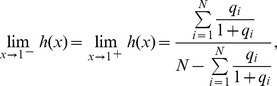


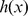
 is a monotonically decreasing function for 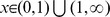, and 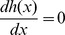 if and only if 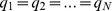.

### ASE Selection

**Figure 6:**
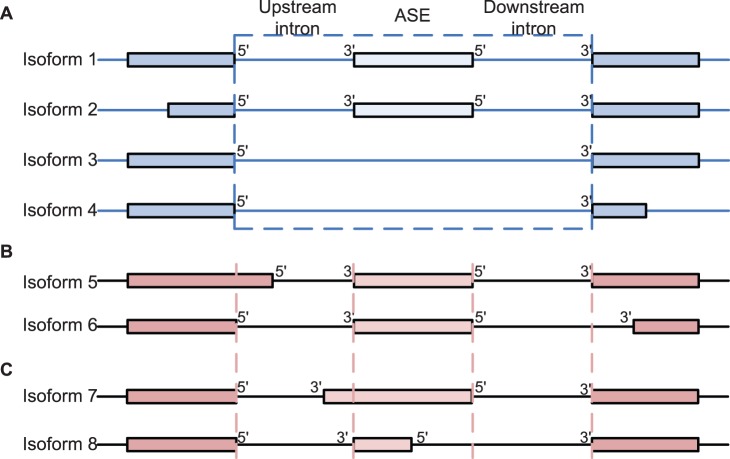
Illustration of ASE selection criteria 3 and 4. (**A**) ASEs that satisfy both criteria 3 and 4. (**B**) ASEs that do not satisfy criterion 3. ASEs in isoforms in both (A) and (B) are not included in our ASE data set. (**C**) ASEs that do not satisfy criterion 4. ASEs in isoforms in both (A) and (C) are not included in our ASE data set.

The KnownGene table of human February 2009 assembly (hg19) was downloaded from the University of California Santa Cruz (UCSC) genome database [39]. We chose UCSC Known Genes as the reference gene annotation, since they contain a comprehensive gene set that is constructed mostly from experimental data in Genbank and Uniprot [92]. For each gene, the KnownGene table gives all known isoforms of its mRNA transcripts. An exon was selected as an ASE in our dataset if the following five criteria were satisfied: 1) at least one isoform includes the exon, 2) at least one isoform does not include the exon, 3) the upstream 5′ splice site is the same in all isoforms, and similarly the downstream 3′ splice site is the same in all isoforms, as illustrated in Figure 6A, 4) the upstream 3′ splice site is the same in all isoforms with the ASE, and similarly the downstream 5′ splice site is the same in all isoforms with the ASE, as illustrated in Figure 6A, and 5) both the upstream and downstream introns are of 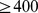 nts. With these strict criteria, the five regions of the ASE shown in Figure 1A are defined without ambiguity. Note that isoforms in Figure 6B do not satisfy criterion 3), and thus ASEs with isoforms in Figure 6A and 6B are not included in our data set. Similarly, isoforms in Figure 6C do not satisfy criterion 4), and ASEs with isoform in Figure 6A and 6C are not included in our data set. To ensure a reliable estimate of the expression ratio, we only kept ASEs with gene expression level greater than 3 RPKM (reads per kilobases per million mapped reads). This gave a set of ASEs for each tissue. The number of ASEs for each tissue is given in Table 1 and more detailed description of the ASEs including their genomic coordinates are given in Table S2. Most ASEs were used for model inference in almost all tissues. Some ASEs were not used in a specific tissue because they did not pass the minimum expression requirement in that tissue. Note that although almost the same set of ASEs were used, the splicing response variable 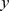 of the same ASE was different in different tissues, and thus the data used for model inference were in fact different for different tissues.

### RNA-Seq Data

The data set in [1] includes RNA-Seq reads from 9 tissues: adipose, whole brain, breast, colon, heart, liver, lymph node, skeletal muscle and testes, as well as several cerebellar cortex samples and cell lines. We only used RNA-Seq data of 9 tissues, which contains over 200 million reads of 32 nts, to detect SREs and cooperative SRE pairs.

### Estimation of Expression Level and Inclusion Ratio

We started by mapping the RNA-Seq reads against an expanded human genome (hg19) downloaded from the UCSC genome database, allowing up to two mismatches, using Bowtie (version 0.12.7) [93]. The expanded human genome consists of the UCSC hg19 whole genome reference sequence and the 56 nt long splice-crossing sequences for each exon junction documented in the UCSC KnownGene table. Reads that could be mapped to multiple loci of the genome were excluded, and 140 million uniquely mapped reads were kept for the following analysis.

We next calculated the expression level of each isoform including or excluding a selected ASE in 9 tissues using the algorithm of Jiang *et al.* [94]. Since we only kept uniquely mapped reads, for an exon of length 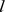, we used an effective exon length 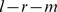, where 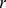 is the read length and 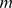 is the number of multi-mappable positions of the exon. To find out 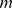, we re-mapped all possible 32-nt subsequences of candidate ASEs and splice junctions against the same expanded genome described above using Bowtie [93]. Moreover, to minimize the effect of non-uniformity of read distribution [95], we only used three exons, including the ASE itself, the adjacent upstream and downstream exons to estimate the expression level of each isoform.

After the expression level of each isoform of the selected gene was calculated, the inclusion ratio (IR) of an ASE in a specific tissue was calculated as the ratio of the expression level of the isoforms with the ASE to the total expression level of all isoforms of the gene, *i.e.*, 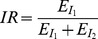, where 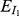 is the total expression level of isoforms including the ASE and 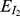 is the total expression level of isoforms excluding the ASE.

### RNA Sequence Elements

For each tissue and each ASE, we extracted all hexamers in five regions around the ASE, including the 200 nts intronic region adjacent to the upstream 5′ splice site (UU in Figure 1A), the 200 nts intronic region adjacent to the upstream 3′ splice site (UD in Figure 1A), the ASE region (EXON in Figure 1A), the 200 nts intronic region adjacent to the downstream 5′ splice site (DU in Figure 1A) and the 200 nts intronic region adjacent to the downstream 3′ splice site (DD in Figure 1A). The EXON region is the ASE itself if the ASE is less than 200 nts; otherwise, it is the combination of the first and last 100 nts of the ASE. Since the 5′ and 3′ splice sites have the consensus sequences MAG/GURAGU and Y
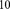
NCAG/G [11], [96], respectively, we excluded the sequences in the window from −3 to 6 around the 5′ splice site and in the window from −14 to 1 around the 3′ splice site in our analysis.

### Variable Screening

Before applying the Lasso, we used a strategy similar to the sure independence screening [40] to reduce the dimensionality of the feature space, thereby improving variable selection in terms of both speed and accuracy. Specifically, for the 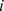
th hexamer, 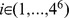, we used the following simple linear regression to test the correlation between its presence in one of the five regions of ASEs with the response variable:
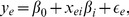
(22)where 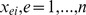 is a binary variable to indicate if the 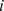
th hexamer is present (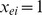) or absent (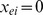) in one of the five regions of the 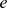
th ASE, and 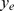 is the splicing response of the 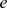
th ASE as defined earlier, and 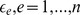 are independent and identically distributed normal random variables. In some samples, we have inclusion ratio 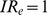 or 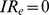, usually due to the low read abundance of the minor isoform. For these samples, we set 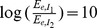 for 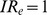 or set 
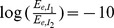
 for 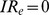, which is equivalent to 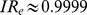 or 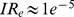. Hexamers having a significant correlation with a p-value 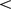 0.05 were kept in set 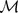 for further analysis with the Lasso and the adaptive Lasso.

In the next step, for each pair of the retained hexamers, their interaction was tested using the model:
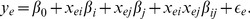
(23)


Interaction terms with a p-value 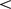 0.05 were also kept in set 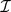 for further analysis. To reduce the possible false positive effects, we also required that the co-occurrence frequency of the two hexamers in an interaction pair was significant (p-value
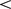
0.05 from a hypergeometric test based on the null hypothesis that the presence of the first hexamer is independent of the presence of the second hexamer) in the five regions of the selected ASEs defined earlier, and that any hexamer or hexamer-pair must be present in at least 1% of the ASEs.

### The Lasso and the Adaptive Lasso

We define 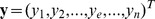 and 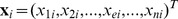, where 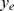 and 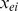 are defined earlier. We also define 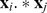 as element-wise multiplication of two vectors. The Lasso procedure was performed by solving the following problem [41]:
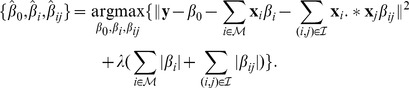
(24)


The optimal value of parameter 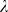 was obtained using 100-fold cross-validation based on the mean squared prediction error. Then we chose 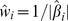 and 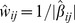 and solved the following adaptive Lasso problem [42]:
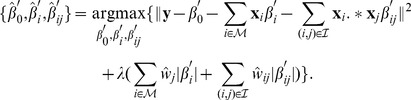
(25)


The optimal value of 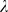 was also obtained using 100-fold cross-validation. We solved these problems using the coordinate descent algorithm of Friedman et al. [97] implemented in the ‘glmnet’ package.

### Refitted Cross-validation

RCV is a technique to estimate residual variance in linear regression models of ultrahigh dimension [44]. In our case, the 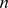 samples were randomly split into two even datasets. We applied the Lasso to the first dataset to select a set 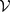 of variables from the variables in 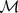 and 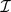 resulted from the variable screening procedure. We then again used the Lasso to refit the model with the variable set 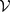 to the second dataset. The refitting process selected a set 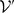 of variables from 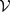. Finally, the variance of the residual error 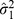 is estimated from the second dataset with variables in 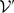 using the OLS method. We reversed the role of the two datasets, and obtained another estimate of the variance of the residual error, 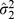. The final estimate is then defined as 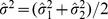. We repeat this process 100 times by randomly splitting the dataset, and the average variance 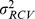 of the 100 estimates was the final estimate of the residual variance.

### Final Model Selection and Correction for Multiple Testing

The adaptive Lasso procedure produced a sequence of models for different values of 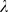. For each 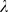, we extracted the variables of the model and estimated the residual variance 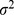 with these selected variables using the OLS method. For the sequence of models, we selected the one having the smallest variance that was larger than 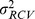 as the optimal model.

Although the adaptive Lasso selected a set of variable in the final model, it did not give p-value for each variable. We then used the OLS method to refit the model and calculated the p-value for each variable. Based on these p-values, we chose the variables at an FDR 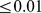 [45]. The final model contained these variables, and the percentage of the variance explained (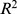) by this model was calculated as:
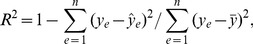
(26)where 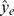 is the predicted value of 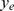 from the final model, and 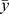 is the sample mean of 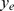. Note that 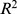 was also used in [26], [34], [38] as the figure of merit for performance evaluation. The software package for model inference framework is available under an open source license.

## Supporting Information

Table S1 The SREs and SRE pairs identified in 9 tissues.
(XLS)

Table S2 The ASEs used in the analysis.
(XLS)

Dataset S1 **The R script implementing the model inference framework.**
(GZ) Click here for additional data file.
